# Uncertainty and the NICU Experience: A Qualitative Evaluation of Family and Provider Perspectives

**DOI:** 10.3390/children10111745

**Published:** 2023-10-27

**Authors:** Katharine Griffin Gorsky, Saloni Butala, Madison House, Chelsea Moon, Sam Calvetti, Tenzin Khando, Michele Kipke, Ashwini Lakshmanan

**Affiliations:** 1 Division of Neonatology, San Francisco School of Medicine, University of California, San Francisco, CA 94158, USA; katharine.gorsky@ucsf.edu; 2 Fetal and Neonatal Medicine Institute, Division of Neonatal Medicine, Children’s Hospital Los Angeles, Keck School of Medicine, University of Southern California, Los Angeles, CA 90089, USA; saloni.butala@westernu.edu (S.B.); madison.house@nyulangone.org (M.H.);; 3 School of Medicine, WesternU College of Osteopathic Medicine of the Pacific, Pomona, CA 91766, USA; 4 School of Medicine, New York University Grossman School of Medicine, New York, NY 10016, USA; 5 School of Medicine, Keck School of Medicine, University of Southern California, Los Angeles, CA 90089, USA; 6 Community Health Outcome Intervention Research, Children’s Hospital, Keck School of Medicine, University of Southern California, Los Angeles, CA 90089, USA; scalvetti@chla.usc.edu; 7 Life Course Intervention Research Network, University of California, Los Angeles, CA 94158, USA; tkhando15@g.ucla.edu; 8 Department of Health Systems Science, Bernard J. Tyson School of Medicine, Pasadena, CA 91101, USA

**Keywords:** transition, NICU, adaptation, uncertainty

## Abstract

There is limited information regarding caregiver and provider perspectives on uncertainty across the continuum of the neonatal intensive care unit (NICU) experience. Open-ended semi-structured interviews were conducted with providers and English- and Spanish-speaking caregivers of infants with a history of admission to a quaternary safety-net NICU. Major themes were generated using inductive–deductive thematic analysis. Seventy-six individuals participated in the study: 47 caregivers and 29 providers. The median gestational age of the infants was 29 weeks and 85% were classified as having chronic complex disease per the Pediatric Medical Complexity Algorithm. Most providers were neonatologists (37%) and nurses (27%) and more than half had over ten years of experience. A conceptual model of caregiver uncertainty was developed and key domains included drivers of uncertainty and its impact, and factors influencing coping and adaptation. Our analysis found a positive association between caregiver information gathering, clinical continuity, support systems, maternal mental health supports, and witnessing a child’s progress and the development of adjustment to chronic uncertainty. These results suggest key areas for intervention that can promote parental adaptation to the uncertainty inherent in the NICU experience.

## 1. Introduction

The experience of hospitalization in the neonatal intensive care unit (NICU) is a profoundly transformative and emotionally demanding journey, both for families and healthcare providers. A cross-sectional study highlighted that the NICU environment, with its abundance of monitors, mechanical ventilators, and constant visual and auditory alarms, creates an unfamiliar and challenging context for parents [1]. It poses unique challenges and emotional complexities, requiring families and providers to navigate a complex web of medical interventions, uncertainty, and emotional strain. Mothers of infants in need of specialized care commence their journey of parenthood in the foreign and often overwhelming setting of the NICU, which may result in delayed maternal attachment [2,3]. The delayed maternal attachment experienced by mothers of infants in the NICU can be attributed, in part, to the feeling of uncertainty that often engulfs parents in this challenging environment. NICU parents frequently express additional challenges, including a loss of control, conflicting emotions of hope and hopelessness, and feelings of guilt experienced by mothers who were unable to carry their pregnancy to full term [4].

As parents navigate the intricate and unpredictable journey of their infant’s medical care in NICU, they are frequently confronted with a profound sense of uncertainty. Uncertainty is defined as “the ability to determine the meaning of illness-related events and accurately anticipate or predict health outcomes” [5]. In a prospective study conducted to explore the relationship between feelings of uncertainty among parents of infants requiring NICU care and the increased risk of developing perinatal post-traumatic stress disorder (PPTSD), parents who screened positive for PPTSD 3 months after hospital discharge reported more uncertainty both while in the NICU and 3 months after hospital discharge [6]. In a study conducted to examine the roles of constructive and dysfunctional problem-solving strategies in the relationships between illness uncertainty and adjustment outcomes in caregivers of children newly diagnosed with cancer, greater dysfunctional problems-solving strategies were associated with higher levels of both uncertainty and poorer adjustment outcomes [7]. Provider uncertainty in the NICU is a prevalent aspect of the healthcare environment. Prognostic uncertainty emerged as a key factor associated with moral distress among neonatologists in a prospective qualitative study conducted to study moral distress among neonatologists working in a NICU [8]. By understanding the intricacies of uncertainty, researchers, healthcare providers, and support systems can develop tailored interventions, resources, and strategies to help individuals navigate uncertainty more effectively.

While there has been significant research on the patient and provider experience in the NICU, there are still some gaps in understanding and addressing the specific issue of uncertainty, which is a significant factor influencing their experiences and interactions in that setting. These gaps indicate a need for further research to delve deeper into the complexities of uncertainty in the NICU setting, including its causes, impact, and potential strategies for support and intervention. While studies have focused on uncertainty during the NICU stay, there is a lack of research exploring how uncertainty continues to affect parents and providers during the transition period and beyond. Additionally, the impact of uncertainty on communication and decision-making processes among healthcare providers in the NICU remains an area that requires further investigation [9]. The objectives of this study were to describe the role of uncertainty (1) during the NICU experience and during the transition-to-home period, (2) accessing community-based services, and (3) addressing mental health.

## 2. Materials and Methods

### 2.1. Research Team and Reflexivity

The authors that conducted the semi-structured interviews were S.B., M.H., C.M., and S.C. The interviews were supervised by the principal investigator, A.L. (a neonatologist). The research team had experience with qualitative research methods. The participants knew the occupation of the research staff and research objectives. The children’s diagnoses and medical history was known to both the participants and the research team.

### 2.2. Study Design and Framework

This was a qualitative study that was cross-sectional. We used an inductive–deductive thematic content analysis as our methodological framework to orient our study. We conceived a conceptual model based in Mishel’s Uncertainty in Illness Theory (UIT) [5].

### 2.3. Participant Selection and Setting

Purposive, typical case sampling was used to recruit caregiver and provider participants at a quaternary urban safety-net children’s hospital between 2019–2021. Caregivers who were >18 years of age or older and English- or Spanish-speaking, whose infants had gestational ages < 37 weeks or required neurodevelopmental intervention were recruited face-to-face or by telephone. To capture a varied range of experiences during transition from NICU to home, caregivers nearing discharge were recruited from the NICU and pediatric wards while caregivers up to 6 months post-NICU discharge were recruited from a high-risk infant clinic. This clinic provided multidisciplinary follow up for infants with gestational age < 32 completed weeks or birth weight < 1500 g or infants at risk for neurologic abnormality. Providers who worked with infants in the NICU, high-risk infant follow-up clinic, or general pediatrics were recruited face-to-face or by email. Study protocol was approved by CHLA Human Subjects Protection Program and all subjects gave informed written consent.

During the COVID-19 pandemic, caregiver recruitment pivoted to the inpatient setting, where caregivers who met inclusion criteria were approached face-to-face by the Principal Investigator (A.L.). In observance of COVID-19 safety measures, caregivers were given information and had the opportunity to ask questions about study participation by telephone and informed written consent was obtained using DocuSign by email and/or text. Provider recruitment experienced no change, but provider informed written consent was also received on DocuSign. We finished recruitment once thematic saturation was reached.

### 2.4. Development of Interview Guide

The development of the interview guide was informed by previous work [10]. After development of the interview guide, it was pilot tested and refined by key stakeholders (families, providers) prior to administration with study participants. Field notes were collected during all interviews.

### 2.5. Data Collection

The data collection involved two groups: providers and caregivers. The caregiver interviews were conducted over the telephone approximately three months after enrollment. The telephone call consisted of a 45 min interview. Providers were interviewed during a 15–20 min telephone call. The interviews were audio-recorded using a digital recorder and sent to the third-party service for transcription. The participants received a gift card for their participation in the study.

### 2.6. Data Analysis

The analysis included descriptive analyses and qualitative analyses. In terms of software, SAS v.9.4 was used for quantitative analyses while Dedoose Version 8.3.35 was used for qualitative analyses. The transcribed audio recordings were uploaded to Dedoose for managing, analyzing, and presenting qualitative and mixed-method research data (2020). Los Angeles, CA, USA: SocioCultural Research Consultants, LLC (www.dedoose.com, accessed on 1 February 2021) [11]. We used an inductive thematic-analysis approach; an iterative process of coding to identify the patterns between concepts. We created codes to help identify recurring themes among the many transcripts. The transcripts were divided between two coders and a third coder double-coded the transcripts to increase validity. We met weekly to discuss progress and adjust the codebook accordingly.

## 3. Results

### 3.1. Demographics

Participant characteristics are captured in Table 1 and Table 2. As shown in Table 1, there were a total of 47 caregiver participants. The median (IQR) gestational age was 29 weeks (26, 34). Most infants (85%) were categorized as having complex chronic disease (C-CD) using the Pediatric Medical Complexity Algorithm [12], with nearly half having sensory issues and global developmental delays, and 38% receiving early intervention. Regarding caregiver demographics, 70% of participants were insured by Medicaid/California Children’s Services, the majority categorized English as being their first language (89%), and 15% lived in a service planning area with a 4th-quartile economic hardship index. In terms of providers, most were physicians or nurses (22% attending neonatologists, 14% trainees, and 35% were nurses) and 69% had more than 5 years of work experience.

### 3.2. Conceptual Model

**Figure 1:**
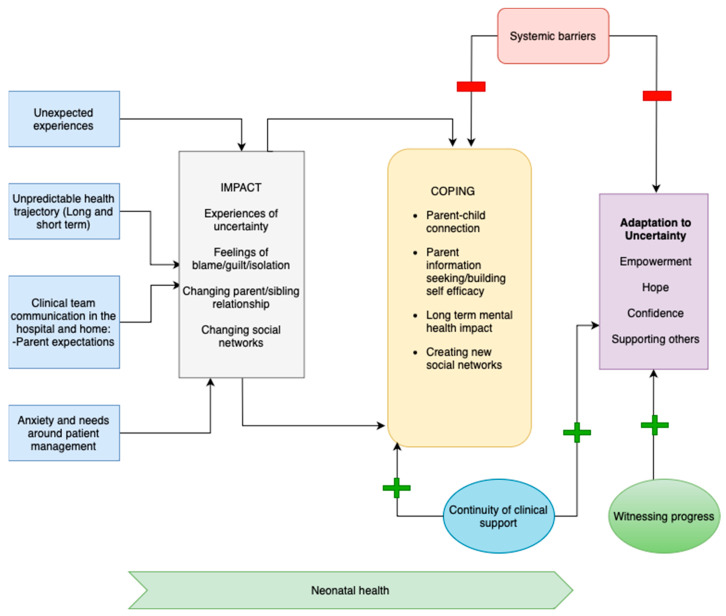
Conceptual Model.

A conceptual model (Figure 1) was constructed to illustrate the process of parental uncertainty in the experience from NICU admission to the follow-up period. This model is based on Mishel’s Uncertainty in Illness Theory (UIT) [5]. In Mishel’s theory, the processing of uncertainty consists of five stages: antecedents to uncertainty, experience of uncertainty, appraisal/impact, coping, and adaptation. The model developed in this analysis adds additional themes of clinical continuity and structural barriers, which were found to impact parental coping and adaptation. The model also seeks to describe the caregiver experience across key timepoints: NICU hospitalization, discharge and transition home, and outpatient follow up. As described in Mishel’s Reconceptualization of the Uncertainty in Illness Theory, chronic uncertainty consists of ongoing processes of appraisal, coping, and adaptation leading to a new equilibrium over time [13]. Thus, this conceptual model should be viewed as an iterative process that builds upon prior parental experiences.

### 3.3. Domains/Themes

The following outlines the domains and themes that were captured.

### 3.4. Antecedents of Uncertainty and Experience of Uncertainty

The themes related to antecedents of uncertainty are captured in Table 3. Parents described experiences of initial disruption followed by feelings of unknown and worry at the time of transition to home. One parent described the feeling that “we were just thrown into these situations…”, emphasizing the unexpected nature of the NICU experience. The feeling of disruption related to the unexpected evolves into an experience of persistent worry in the setting of unknown health trajectories. As described by one caregiver, “It was the thing that scared me most of the time was the fact that we knew there was something with him, but we didn’t know exactly the long term … That was the scary part, like not knowing exactly what we were facing …”. This feeling was echoed by many caregivers, describing fear that their child may, “stop breathing on the way back home” and “always knowing in the back of your head like this could happen. So watching out for signs of that was the scariest part”.

This worry and uncertainty surrounding the transition home was often fueled by feelings of anxiety related to the management of complex medical needs and a sense that “once you get home, you’re by yourself”. Many caregivers described the stress of transitioning from a space of constant support to one where “you’re doing the job of the respiratory therapist, two nurses, the everything. And you have to really know your baby and be able to recognize signs if something’s wrong”.

The unpredictable health trajectory led to a description of persistent uncertainty, often punctuated by disruptive interactions with the health care system. “Sometimes I feel like my world will stop, like when he gets even admitted to [the hospital], from this day, it was like my day, my life is just on hold until he actually gets better within time. Oftentimes I feel like he’s not gonna get better when he gets older because everything that he’s diagnosis is a little more chronic. So I can’t seem to get over that he might be better with the time or he might not”. The initial NICU hospitalization acts as an antecedent for uncertainty, which evolves into a chronic state of unknowing as children continue to grow and their health trajectory evolves.

Provider interviews also emphasized the experience of caregiver uncertainty in the NICU and the need to explicitly address this uncertainty as a means to support caregivers. One provider stated “I think the despair that people can feel about, ‘My baby is really sick. I don’t know what will happen. There’s so much uncertainty’. I think addressing that uncertainty is something that could be done more specifically, to help us take care of the family too”. Another provider echoed this sentiment, describing the importance of the “normalization of the things that parents experience, like the significant rollercoaster and constant uncertainty” as well as having, “something to address that might be helpful as an adjunctive to things like family meetings and social support from the social worker in the unit or something”.

The experience of caregiver uncertainty was impacted by team communication and expectation setting. Caregivers emphasized the importance of honesty in provider communication: “If they didn’t know at the time, they were also honest about that… We rather people tell us they don’t know than for them to tell us something, and then later on not be true…”. One caregiver described coming to a realization of “how much art there is in medicine. I thought it was a lot more science. It was really interesting and eye-opening that we can have dialogue and have discussion about what was going on and what their thought was, and to know that certain things they didn’t know the answer for, it was kind of trial and error”.

Caregiver uncertainty was worsened when the medical team’s communication and expectations eroded parental agency. One caregiver described a feeling that the team saw their baby as “a number” and that this “led to quite a few instances of stress, where they would decide to do something that isn’t something we discussed. …. And then I would show up and they would be like well we want to do this and this. And I’m like no, we’ve already talked about it, I don’t understand why I’m having to like say this again. Those things were very frustrating”. Some caregivers also described limited expectations for parental involvement in cares, stating that “we weren’t allowed to do a whole lot. … When we were finally able to go home, I didn’t know what to do because I was never allowed to do anything”. This also caused distress in transition to a step-down unit prior to discharge home. In a new unit, caregivers were faced with shifting expectations: “we’ve never done this before, so we will need a little instruction now, and figuring out the expectations, in the NICU, where there are things you’re not allowed to do versus upstairs where there are things there that you’re required to do”.

Providers also emphasized the importance of caregivers as decision makers. A provider recognized that “The parents [are] the best historian. They know something that’s changed in their child that isn’t necessarily noted in the doctor’s note. …. The parents have a unique perspective on their child’s well-being that, as medical providers, sometimes we’re just not in tune with”. Providers also described the challenging feeling that can come when the communication regarding an infant’s health trajectory is constantly in flux. The lack of explicitly addressing uncertainty can result in feelings of frustration. “‘Yesterday you said that it was fine. And now today, you’re saying it’s not fine. And that doesn’t make sense’. And parents feel like things are going up and down all the time, it’s like, ‘Well, this can’t be the way that this is supposed to be’”.

### 3.5. Impact

Caregivers described feelings of guilt, blame, and isolation in response to the uncertainty fostered by the NICU experience, as outlined in Table 4.

“They used to tell me he was doing good, he was a healthy baby, but I was just blocked in that part… I started blaming myself, probably I put him through this for waiting this long to have a vaginal [delivery]… Then the doctors keep explaining it’s not your fault… So I was just blaming myself for everything that was going on, that I wouldn’t let nobody tell me this is correct, this is the right thing”.

“I just felt very disappointed in myself. I just felt like I couldn’t do anything well, and I couldn’t do anything right, and that I wasn’t the mother who I imagined myself to be, and I just wasn’t the partner, I was failing at everything”.

“I couldn’t concentrate at work, but work was kind of the only thing that I was still able to do okay at, because I didn’t talk about anything there. So nobody even knew what was going on. And not having maternity leave, like I was pregnant, and then I wasn’t, but nobody knew. And it was just very weird, and I just kind of isolated myself”.

The isolation felt by caregivers was reflected in descriptions of altered social networks and a shift stemming from an unexpected and uncertain event. Many described a feeling that people didn’t know how to support them. One parent reflected that, “I think when you lose a child, there is nobody that you can, like, I don’t know. It just was very isolating… And people don’t know how to talk to you and nobody really knows how to support you”. The lack of support from social networks continued into the post-NICU experience, exacerbated by the uncertainty related to the COVID-19 pandemic and a child’s ongoing medical needs.

Provider perspectives on social networks differed slightly from those of caregivers. Multiple providers described the overwhelming number of requests that parents received for updates on the child’s status and suggested tools for addressing this: “So one of the things that I always suggest is to have an outgoing message, basically updating people, giving the information that you are comfortable giving for an update”. Many providers also commented on navigating the impact that the NICU stay might have on older siblings. One provider stated “… quality is more important than quantity. In other words, spending time with each child individually and talking, letting them talk about themselves, not necessarily about the baby, unless they want to talk about. You know but really focused attention, even if it’s for short periods of time it can do wonders”.

### 3.6. Systemic Barriers

As summarized in Table 5, caregivers raised multiple systemic barriers that impaired their ability to cope and adapt to uncertainty. One described that they “had the biggest issue trying to get her prescriptions because of the insurance”, due to prior authorization needs, and that “it was just a whole complicated mess that upset me ‘cause that was a waste of time, twice”. Others expressed difficulties getting scheduled for follow-up appointments: “Scheduling is the hardest thing, because I know that everything is so backed up all the time. The high-risk infant clinic took us months and months to get in. … And then just the referrals for insurance, it takes a while”. Another reported that, “The most challenging thing was transportation, driving to LA”.

### 3.7. Continuity of Clinical Care

The continuity of clinical care, both inpatient and outpatient, emerged as an important theme impacting parental coping and adaptation, as reviewed in Table 5. Many caregivers emphasized the value of continuity within the inpatient clinical care team, particularly that of primary nursing. They expressed that this provided support, learning opportunities, and a sense of comfort with the NICU process.

“I actually had primary nurses, and I think that that was really helpful because they really got to know [child M.] and I really got to know them. … having that consistency, I think was really beneficial for myself and for the baby. And probably for the staff too, because they felt comfortable and they knew her. If she’s having an off day, or something, because they were able to pick up on those things, it meant that I was going to be able to, too. So, me being consistently with her, I was gonna learn stuff about her and that would help make everything more smooth”.

Parents also voiced frustration with provider turnover, stating that “having a new resident every time was very frustrating, because it was like you don’t know my baby. And if you’re making these decisions, I’d appreciate that we have a conversation about this, so I can catch you up”.

The value of clinical continuity continued into the outpatient space where many children required multiple sub-specialty appointments and connections with developmental services. One parent reflected that, “There’s a lot of appointments and a great team of folks that talk to each other. When I go from one appointment to the other, they already know what one or the other has been talking about, [which] has been very helpful…. It makes me confident [in] the work that they’re doing, and it keeps me at ease knowing that he’s in good hands”.

The transition from the NICU to home stood out as a period in which clinical continuity greatly impacted parental feelings of uncertainty. Many parents felt that having access to a provider for questions would be a helpful resource. One parent stated that it would be helpful to have “a number that you could call somebody 24 h a day. Because like my pediatrician, he’s only available weekdays Monday through Friday” to avoid having to take their child to urgent care and be exposed to sick children. Another reflected on the potential benefit of “having the resource to reach out to nurses or physicians that would get back to you quickly, and for nonurgent things. How do I make sure this dressing is correctly applied? Things like that. Cause if you reach out to your doctor, you know that could take several days for them to get back to you”. Multiple caregivers commented on the value of being connected to developmental services prior to discharge: “I think it’s very important. For the NICU to make that first contact with the regional center for us was really important, cause it’s hard to do that”.

Many providers also commented on the value of care coordination and check-ins to support families in the transition from the NICU to home.

### 3.8. Coping

Parent empowerment and self-efficacy through active information gathering was a consistent theme that emerged within the domain of coping with uncertainty (Table 6). Within the NICU, parents emphasized the importance of engaging the clinical team (nurses, NPs, and doctors) through question asking: “I asked a lot of questions the whole time that he was in the hospital. Honestly, that’s where I got the bulk of my information”. Parents felt most supported by clinical teams that expressed an openness to providing information: “All of the people that were there were really supportive and very engaged in ensuring our success and providing a lot of support, not just with resources, but also with the openness saying you have any questions, please feel free to ask them. And when we asked, they were answered”. Additionally, parents described the importance of hands-on learning related to infant care (e.g., bathing, feeding) and more complex medical skills (e.g., Gtube management, providing medication) to their management of uncertainty prior to discharge. A parent stated: “Two weeks before I left the hospital they let me do it by myself. They just went and watched me and [brought] me medicine. That was helpful because when I’m [here] at home, nobody else [is] watching me so I got to learn how to do it”. The importance of active engagement in parental learning continues into the outpatient experience. “Even coming home, I still have the support because even when I go to his appointments, I can always ask these questions over and over again, and they’ll always answer these questions for me in a way that I understand and give me all the information I need”.

Provider perspectives also emphasized the importance of parent engagement. A nurse reflected on barriers to parental engagement in information gathering: “Being a bedside nurse, I would hear it from the doctor, what they said. And you can see them nodding and nodding. And then, afterwards when they leave, and I’ll say, ‘So did you understand what the doctor said?’ They’re like, ‘I think, but I had a question’. I was like, ‘Oh, my gosh. Why didn’t you say anything’, because they feel they’re just bombarding the doctors, and like their time isn’t worth it”. One provider suggested caregiver access to videos of more complex medical procedures to improve confidence at home.

Parent mental health challenges, specifically those of mothers, emerged as an ongoing process that manifests in response to the emotional impacts of uncertainty. One mother reflected on the ongoing nature of her coping process—“I am a work in progress”. Another emphasized that “It doesn’t just go away. … It’s a much longer trajectory I think you’re dealing with things, and they manifest in different ways”. Mothers also emphasized the importance of recognizing mental health needs and the value of support from the clinical team (social workers, psychologists, nursing, etc.) and family: “I do think that having more specialized care and understanding maternal mental health is something that is just a huge gap, and I think that it’s really important that we do a better job with that. … And I think that we really need to look at it as if it goes untreated, you’re dealing with that forever until it is treated”. Multiple mothers described feeling like their mental health struggle “wasn’t acknowledged and it wasn’t met with any services”. While some mothers felt a “peace of mind” and that they were “in a better place now” after speaking with a mental specialist, others expressed that they, “didn’t feel supported through the follow-up appointments. I didn’t feel supported from the hospital necessarily. …‘cause what I’m dealing with, and still dealing with, is some maternal mental health issues, and PTSD”.

Providers also acknowledged the importance of ongoing maternal mental health supports, emphasizing the value of inpatient screening and connection to services and that they “hope they [the supports] continue that once they leave the hospital. I don’t know how much follow-up [there is] afterwards”. Many providers also brought up the need for parents to engage in self-care. One provider stated “Conveying to parents that you, at some point you need to take care of yourself alone because you should be healthy and maybe you have other kids and just to get through this you need to be healthy and strong. But it’s also important because it does impact the child”. This was occasionally reflected in caregiver perspectives regarding their coping process. One stated, “I had that constant update and reminder all the time from all the nurses, like take some time for yourself. And I appreciated that”.

Caregivers emphasized experiences that enabled them to bond with their infants as key components to help cope with the NICU experience.

“One really helpful component for us was music therapy. We’re both really musical and we couldn’t hold him for the first month and that [14] hard. I think we kind of built that bond through music …We had so many people looking after the medical side of things, but that really helped us build that connection to him when we couldn’t especially when we couldn’t cuddle him… So I think that really helped us like learn how to connect with him”.

Other parents touched on the value of books to read to their infant and ways to promote parent–infant bonding while in the intense medical space of the NICU.

Social support from multiple sources—family, friends, social media, other NICU families, and religion—was an essential factor for caregiver coping. Beneficial support from family and friends often came in the form of practical support such as GoFundMe campaigns, childcare for older siblings, and providing meals. A caregiver expressed gratitude for the financial support organized by a friend through a GoFundMe while their household was limited to one income. “People banded together and helped our family in that way. That really helped us to be able to cover rent”. Many also referenced the support from friends and parents who could care for older children at home and make meals for the family. One parent described how her friend, “just came in and didn’t need anything from us, and made us food, and bought us dinner… She helped streamline things and just made things really easy while we were going to the NICU”. Another commented on the value of support from her mother, commenting, “it’s hard enough with one medically complex kid. Then adding a three year old at the time just made it a little bit more crazy. She decided to stay with us until we got our feet up from under us”.

The support of caregivers with similar experiences provided an important community for the caregivers interviewed. They expressed the value of receiving support from people with similar experiences, a comfort in finding community, and a sense of reassurance regarding their child’s situation. One parent described the difference between support from the clinical team and from other parents: “It’s parents with experience. They’re going through the same thing that happened with you, not a professional who’s only dealt with it by reading… they know what they know, and they’ve put their time into knowing and trying to help others. But I guess it’s different having the knowledge, and having lived experience”.

Providers echoed the importance of connecting caregivers with a community of support from those with current or prior NICU experience. One reflected on how “The NICU’s really overwhelming”, and that often, “most of the parents sometimes feel like they’re very alone in the NICU and they don’t have anybody to talk to, especially if they don’t have support within their family”, and that “if they’re going through something specifically and then you find somebody else that is going through that as well, I feel like you, the parent might potentially feel that they can talk to somebody…so I think [that is] very useful”. Many recommended creating programs and structures that facilitated connections with prior NICU parents, as well as ways to foster community amongst parents within the NICU.

### 3.9. Adaptation

Despite ongoing challenges, many parents expressed finding a new equilibrium and acceptance of uncertainty in adaptation (Table 6). One parent reported that, “I think my comfort level with caring for him, and seeing his growth, and just his personality come through, like seeing him grow. Besides the oxygen and the G-tube that he’s connected to, and having to go to so many doctor’s appointments, he is a regular baby”. Many other parents echoed these feelings of increased confidence in both their abilities as caregivers but also in their child’s ability to make progress: “I just was feeling very confident that when she came home, that she would thrive, like being home is what she needed to do”. The value of witnessing their child’s progress and the hope and joy that this created emerged as a theme within the domain of adaptation. One parent described the progress their child made with oral feeding: “Now he can eat, and he still doesn’t eat a lot. But the fact that he can eat and not choke is amazing. Just keep loving on that baby, he’ll surprise you”. This experience was echoed by many parents who witnessed progress in their child’s ability to feed and the joy in seeing that progress.

Caregivers reflected on the value of being able to support other parents and finding a sense of empowerment and purpose through this connection. One mother reported that connecting with a support group helped her recognize that, “we’re all kind of dealing with these traumas, and what does that mean, and it gives me something to focus on. I don’t feel hopeless, because I feel like there are things that we can do to change these systems and make sure that this isn’t the way that people are left feeling”.

Providers also commented on the value of witnessing progress. One reflected that, “I think sometimes we focus a lot on all the medical problems that the baby has and kind of lose sight, and I think the providers are guilty of this too, lose sight of the developmental progress that the baby should be making at any given age, and how to support the baby as best we can”.

## 4. Discussion

This study is among the first to assess the evolution of parental uncertainty from the NICU stay through follow-up care in the outpatient setting. Our analysis aligns with prior work that established parental uncertainty in illness as a key theme throughout the inpatient NICU experience [15,16]. Our study also incorporates provider perspectives on parental experience of uncertainty, which offers additional opportunities to consider how to address the parent experience at a unit and system-wide level. We built upon Mishel’s Reconstitution of Uncertainty in Illness Theory to establish a conceptual model that tracks the initial experience of uncertainty as it evolves through the phases of impact, coping, and adaptation. In her reconstitution of uncertainty in illness, Mishel describes a process by which parents establish a new equilibrium that accepts and adapts to uncertainty [13]. Our data reflect this theory, demonstrating that the cycle from uncertainty to adaptation is iterative as parents face new challenges (e.g., transition to home, new illnesses, etc.) and adapt to a new equilibrium. For many parents, this process resulted in a positive adaptation to chronic uncertainty, in which they described feelings of confidence and hope. However, for others, a lack of positive coping mechanisms led to an ongoing sense of worry and fear that fueled persistent trauma and anxiety. This reflects prior findings by Malin et al., which demonstrated that ongoing negative associations with uncertainty are strongly associated with higher levels of PTSD and anxiety [6]. Given that chronic uncertainty is unavoidable in the setting of complex illness, the data from this study provide insights into ways that the healthcare system can support a positive adaptation and adjustment. Below we map out the findings within the conceptual model over its iterations to identify key areas for intervention.

The nature of uncertainty experienced by families evolved over the course of the NICU hospitalization, the transition to home, and into the outpatient setting. Initial descriptions featured elements of shock and significant prognostic uncertainty. As families transitioned to home, we saw that parents described their experience of uncertainty with words such as “worry”, “alone” and “scary”, reflecting both an ongoing evolution in the child’s health trajectory as well as a significant shift in the amount of immediate support and monitoring available. These feelings sometimes continued in the form of hypervigilance to the infant’s behaviors, while for others there seemed to be an acceptance of uncertainty and focus on the infant’s progress. An analysis of uncertainty during the NICU stay by Krick et al. found that parental uncertainty shifted from feelings of initial shock to what they describe as a “Gray Daze” and finally into “Looking Forward”, a progression which they summarize as a shift in a view of uncertainty as changing, “from something to be feared to something that was accepted and integrated into their view of the future” [15]. We found similar results in our study—demonstrating that the experience of uncertainty can vary across situations and in response to prior experience.

While the experience of uncertainty varies, our analysis makes it clear that uncertainty is unavoidable in the setting of a NICU stay and its sequalae. Providers commented on the importance of openly discussing uncertainty with families and supporting families in the management of ongoing prognostic uncertainty. When providers engaged in open communication regarding clinical uncertainty, families reported a greater acceptance of uncertainty and an ability to move forward. This mirrors the results from an analysis of surrogate decision makers’ preferences in communication regarding a patient’s critical illness, which found a strong preference for an explicit acknowledgement of prognostic uncertainty and that this built trust in the clinical team [17]. Explicit discussions of uncertainty present an opportunity to normalize challenges of the NICU experience and to build connections with caregivers.

The uncertainty that surrounds the NICU experience had a significant impact on parents’ emotional well-being and their relationships with others. Prior research suggests that a major disruption in the parental role and meaning system occurs as a sequalae of the NICU admission [18]. Parents proceed through pregnancy with certain expectations for what parenting will entail, a vision which becomes dramatically disrupted by the NICU experience [18]. This disruption results in feelings of self-blame, guilt, and isolation as parents struggle to comprehend the origins of their current situation. The significant deviation from the expected postnatal experience also impacts the parental social network. Parents described challenges interacting with their prior social networks when the supporting individuals struggled to process the events themselves. Parents also commented on the challenge of caring for and supporting the infant’s older siblings. Many providers noted that siblings may also have feelings of guilt and isolation, and the importance of incorporating them into the NICU experience.

A deviation from the expected parental role continued into the period of transition from the NICU to home. Specifically, the expectation for parental involvement in their child’s care shifted dramatically, from a situation in which the majority of the care and monitoring was performed by clinical providers to the majority being performed by parents. Many caregivers described this transition as scary and emphasized the feeling of being alone. The feeling of worry was exacerbated by a new feeling of ambiguity as to what types of behaviors or changes in their child signified a clinical concern as opposed to an expected change. As suggested by White et al. in their analysis of uncertainty in the transition period, parents would benefit from greater support and explicit planning around uncertainty to minimize the emotional impact from this significant transition [19].

As described in Mishel’s Uncertainty in Illness Theory, impact is followed by a process of appraisal and coping [5]. Parents appraise the uncertainty as a danger or opportunity, and subsequently adopt respective coping mechanisms. Coping ultimately leads to an adaptive or maladaptive state, hinging upon the parental appraisal of uncertainty. As previously discussed, this process often reoccurs over time, as parents constantly reappraise their situation and modify their coping approach. Uncertainty that may have initially been perceived as dangerous can evolve into a scenario of hopefulness and optimism. In our analysis, we saw that coping mechanisms and the factors that influence coping often support this shift. Although uncertainty initially led to feelings of guilt and isolation, over time parental engagement, social support networks, mental health supports, witnessing progress, and clinical continuity promoted feelings of hope, confidence, and self-efficacy among many parents.

In this analysis, we approached parental coping using the social ecological model as a conceptual framework. The social ecological model describes individual behaviors within graduated levels of social and environmental contexts with which the individual interacts. The model has been applied to describe caregiver adaptation to childhood developmental delay and childhood cancer [20]. In this adaptation, we considered five levels: the individual (parent), interpersonal relationships (parent–child, family, etc.), neonatal ICU Community (NICU provider teams and processes), health system (Inpatient and outpatient, insurance, community programs), and public policy.

At the individual level, key coping practices involved information gathering and mental wellbeing. Many parents described an active information-gathering process through asking questions and participating in their child’s care. For many, this approach to knowledge acquisition continued into the outpatient setting as well. Approaching uncertainty through an active engagement in learning fostered confidence despite ongoing complex medical needs at home. Importantly, this learning was supported by the NICU community, which provided parents with opportunities to engage in care and in medical discussions.

The interpersonal level featured key relationships between the parent and child as well as the parent and their social networks. Parents reported witnessing progress in their child as a key factor promoting parental adaptation to child illness. In an analysis of experiences of caregivers of children with Trisomy 21, Truitt et al. found that hope was the most significant factor predicting adaptation with perceived uncertainty as a separate independent predictor [21]. In our study, parental ability to bond with their child and bear witness to positive growth both fostered hope and mediated the impact of perceived uncertainty.

Support from social networks also assisted with adaptation to uncertainty. Financial and practical supports from the parental social network allowed parents to focus their energy on coping as opposed to worry related to financial stressors and childcare. Parents also described important relationships with other NICU families and parents with prior NICU experience. These relationships occurred in person, over social media, and through other connections. For many parents, hearing the experience of others who had been down this path provided reassurance in the face of uncertainty and promoted positive coping behaviors.

The practices and policies at the NICU level impacted parental coping and interacted with all levels of the ecological model. As described above, family-centered communication that empowered families to ask questions and engage with the medical team supported parent confidence and knowledge building. Additionally, primary nursing as a unit policy promoted clinical continuity and decreased parental stress through building trust with clinical providers. Direct and hands-on teaching prior to discharge, as well as establishing connections between parents and outpatient resources (e.g., early intervention and the local school district) enabled parents to feel better prepared for the transition to home. Screening for and addressing mental health needs at the unit level assisted in connecting families with resources, although often failed to meet ongoing needs.

At a greater health systems level, the clinical coordination of care and systemic barriers impacted parental coping and adaptation. Both providers and parents commented on the importance of care coordination within the outpatient setting, both between the subspecialty and primary clinics but also between parents and different providers of medical equipment and other medical needs. Parents expressed appreciation for individuals who helped them navigate the complex landscape of durable medical equipment and insurance. Others reported appreciation for programs such as Early Intervention, which helped them gain tools to support their child’s development. Parents also reported many challenges at the health system level. Some described difficulties obtaining timely appointments and medications, and many expressed a desire for a provider who could assist with medical concerns over the phone. A lack of ongoing mental health support also impacted parents who described difficulties managing their child’s care while also struggling with symptoms of PTSD.

This study is one of the first in the literature to describe uncertainty in the NICU experience across the continuum from admission to the outpatient experience, and is the first to incorporate provider perspectives. The study is also one of the only such papers to focus on a primarily low-income population. Despite these strengths, the study has several limitations. Our study population was recruited from a single center with a focus primarily on those patients receiving Medicaid, potentially limiting the generalizability of our results. Those with private insurance may have differing access to services and reimbursed costs. Recruitment efforts focused on low-income non-Hispanic Black and Hispanic caregivers, which may have resulted in perspectives more specific to the lived experiences of the individuals in these groups. However, given that many prior studies on parental uncertainty have tended to sample White-identifying caregivers, the predominant non-Hispanic Black and Hispanic population adds to the breadth of the current literature. Moreover, the clinical conditions of the infants and the parents perspectives were heterogeneous, which may have impacted the study results although we sought to represent diverse experiences. Lastly, given the retrospective nature of the study, the results are vulnerable to recall bias.

Recommendations for intervention.

Based on the findings described in the previous sections, we make the following recommendations to support parents through the NICU experience.

Individual:

Family-centered communication: encouraging parents to join rounds, providing space for parents to ask questions, explicitly discussing prognostic uncertainty with families;

Provide tangible resources to promote ongoing parental learning: videos of how to manage medical equipment, classes related to infant care and clinical management prior to discharge.

Interpersonal:

Provide parents with tools to track developmental and medical progress;

Create parent support groups and gatherings within the NICU;

Develop a role of Parent Navigator—someone who has prior NICU experience that can meet with parents in the NICU;

Provide parents with connections to larger support groups via social media.

NICU Policies and Processes:

Develop structures that promote primary nursing for long-term admissions;

Ensure mental health screening of all primary caregivers and connect to resources;

Hire adequate mental health support professionals to support parent needs;

Connect families with outpatient developmental programs prior to discharge to limit parental burden;

Ensure that prior authorizations are completed and all equipment is ready at the time of discharge.

Health System:

Establish a Transition-of-Care Navigator who assists parents in obtaining contact information for clinics, assists with coordination of appointments, and performs a check-in call one and two weeks following discharge;

Enable medical record sharing between Electronic Medical Record systems to ensure communication between teams;

Establish mental health support programs for NICU parents in the outpatient setting.

Public Policy:

Ensure funding for outpatient mental health support for NICU parents;

Establish clinical coordinators within Medicaid and CCS to assist with care coordination;

Increase funding for outpatient developmental support programs such as a Regional Center to ensure access;

Reimburse clinicians for telemedicine visits and care coordination work.

## 5. Conclusions

In this study, we developed a unique conceptual model of parental uncertainty in the NICU experience that draws upon prior theories in the field to illustrate the complex and evolving nature of parental uncertainty in the face of neonatal medical complexity. Through the unique use of parent and provider perspectives, we identified several ideas to support parental coping and adaptation from the individual to policy level. Uncertainty is an unavoidable and ongoing component of the NICU experience; however, improved care coordination, parental empowerment, and increased mental health services may promote positive coping and adaption among parents.

## Figures and Tables

**Table 1:** Infant and parent characteristics (n = 47).

Characteristic	N (%) or Median (IQR)
Infant Characteristics	
Birth weight (grams), median (IQR)	2155 (1122, 3093)
Gestational age (weeks) at birth, median (IQR)	29 (26, 34)
Diagnoses at index hospitalization (neonatal), may have more than 1	
Bronchopulmonary dysplasia, chronic lung disease	17 (36)
Retinopathy of prematurity (requiring intervention)	13 (28)
Necrotizing enterocolitis (surgical)	3 (6)
Intraventricular hemorrhage (Grade 3–4)	12 (26)
Congenital heart defect	23 (49)
Medical complexity (Post-discharge) *	
Complex chronic disease (C-CD)	40 (85)
Non-complex chronic disease (NC-CD) or non-complex non-chronic Disease (NC-NCD)	7 (15)
Require prescription medications after discharge	31 (66)
Require durable medical equipment after discharge	19 (40)
Oxygen	13 (28)
Feeding (gastrostomy/jejunostomy/nasogastric)	11 (24)
Diagnosis of developmental issues (language delay, global developmental delay, motor delay)	21 (45)
Early intervention services received	18 (38)
Self-Reported Race/Ethnicity	
Hispanic	10 (20)
White Hispanic	3 (6)
Black non-Hispanic	6 (13)
White non-Hispanic	12 (26)
Asian	4 (9)
Self-identified as Multiple Race/Ethnicity	12 (26)
Insured by Medicaid/California Children’s Services	33 (70)
Family/parent characteristics	
Primary language	
English	42 (89)
Spanish	4 (9)
Other	1 (2)
Residential zip code in 4th quartile of economic hardship of service planning area	7 (15)

* Defined by Pediatric Medical Complexity Algorithm (version 3.0) [12].

**Table 2:** Characteristics of participating providers (n = 29).

Characteristic	N (%)
Area of practice	
Attending Physician (Neonatologist)	6 (22)
Attending Physician (Pediatrician/High Risk Infant Follow Up)	1 (3)
Nurse Practitioner	2 (7)
Registered Nurse (Neonatal)	10 (35)
Clinical Psychologist	1 (3)
Research Faculty	1 (3)
Clinical Case Coordinator (inpatient and outpatient)	2 (7)
Lactation specialist	1 (3)
Social Worker	1 (3)
Trainee (Neonatal–Perinatal Medicine Fellow)	4 (14)
Years in practice	
<5 years	9 (31)
5 to <10 years	11 (38)
>10 years	9 (31)
Experience with use of a smart device	29 (100)

**Table 3:** Themes and illustrative quotations, antecedents of uncertainty.

	Patient	Provider
Antecedents of Uncertainty		
Unexpected experience	“I don’t feel like you’re getting to a high-functioning person who deals with anxiety, and needs to function, like we just were thrown into these situations, where like you just have to keep functioning. So I’m not hopeless, but I am lost, and I do need help. “But after the NICU, come home, I was so worried because, I didn’t know how, it’s my first baby. So I had to handle with that part, you know, learning how to be a mom. But, being home with the baby with this situation, it was a lot [emotions], a lot of questions, cause in the hospital I had the nurses if I had a question on the baby were acting or doing. But once you get home, you’re by yourself”. I feel like the biggest worry that we had was once we got home [was that] we’re not gonna have a lot of help in terms of like, at the NICU, we have the nurses, we have multiple staff members, where if something happens, it’s not on us. And then coming home, we have another kid to take care of. So it’s like can we provide him with enough attention to be able to get him caught up as much as we can? And then on top of that, making sure our daughter gets what she needs in terms of attention and all the nurturing and all that she needs as well”.	
Unpredictable Health Trajectory	“It was the thing that me scared most of the time was the fact that we knew there was something with him, but we didn’t know exactly the long term, and the care, and it kept coming up, and everything. That was the scary part, like not knowing exactly what we were facing, because we still had to see the specialist, and we still had to do the follow-up appointments”. “It was exciting but at the same time it was scary, because since he was having difficulty with breathing. … So then we were so excited to bring him home because we were waiting for that day, you know, to bring him home and everything. But all the way from the hospital to our house, we were just like is he breathing, is he breathing? It was just scary to think that he would stop breathing on the way back home. So it was exciting but it was scary too to see. They told me he was fine and everything now, but it was just scary for us…” “We’ve left knowing his MRI was clean and everything was good. … But always knowing in the back of your head like this could happen. So watching out for signs of that was, was the scariest part for like, you know he was clenching his fists, so does that mean something. … Like am I supposed to worry about this or is it something normal. So just his developmental was probably the most, just watching out for that was hard”. “Sometimes I feel like my world will stop, like when he gets even admitted to [the hospital], from this day, it was like my day, my life is just on hold until he actually gets better within time. Oftentimes I feel like he’s not gonna get better when he gets older because everything that he’s diagnosis is a little more chronic. So I can’t seem to get over that he might be better with the time or he might not”.	“I think that normalization of the things that parents experience, like the significant rollercoaster and the constant uncertainty I think that people feel, like, “What will my baby be like? What will it be like at home?”, “Will my baby be able to go to school normally,” and those kinds of uncertainties. … But having something to address that might be helpful as an adjunctive to things like family meetings and social support from the social worker in the unit or something”. “I think the despair that people can feel about, “My baby is really sick. I don’t know what will happen. There’s so much uncertainty”. I think addressing that uncertainty is something that could be done more specifically, to help us take care of the family too”.
Team Communication/Information provision	“… are you just coming in here and looking at my baby as a number, and looking at the charts, and then making decisions, or setting goals based on that? That to me was really not helpful either. It led to quite a few instances of stress, where they would decide to do something that isn’t something we discussed. … And then I would show up and they would be like well we want to do this and this. And I’m like no, we’ve already talked about it, I don’t understand why I’m having to like say this again. Those things were very frustrating”. “I thought that when we got the news that we got the scans of her brain, and the doctor who was in charge at the time, she had to meet with us. And she cried, and she was amazing, and that was very helpful, and very human. She was just like I’ve never seen bleeding so bad in any living, especially in someone so young. So when she was delivering that news, she delivered it in a very human way”. “If they didn’t know at the time, they were also honest about that…We rather people tell us they don’t know than for them to tell us something, and then later on not be true, which for me as the most part, it felt like something that happened at other hospitals and that didn’t necessarily happen here”. “I definitely think that going to rounds is very valuable. Having doctors who are willing to talk with you and explain things to you is super helpful. … And I didn’t really realize how much art there is in medicine. I thought it was a lot more science. It was really interesting and eye-opening that we can have dialogue and have discussion about what was going on and what their thought was, and to know that certain things they didn’t know the answer for, it was kind of trial and error. Which is, for someone who hasn’t really had much interaction with the hospital outside of a regular doctor visit, was really eye-opening”. “You get pamphlets and then you’re sent home, but it would have been nice to have a one-on-one with her doctors, so I could know exactly everything that was going on with her, ‘cause there was stuff that I wasn’t aware of… It would have been nice to have the whole team, pulmonary and cardio, whatever, just sit down and go through everything, like you’re gonna be coming back to see us, and these are the issues that you’re gonna have. And some more of [an] explanation. That’s what I wish would have happened”.	“’Yesterday you said that it was fine. And now today, you’re saying it’s not fine. And that doesn’t make sense’. And parents feel like things are going up and down all the time, it’s like, “Well, this can’t be the way that this is supposed to be,” you know, “This isn’t normal. You guys are not doing it right”. “The parents [are] the best historian. They know something that’s changed in their child that isn’t necessarily noted in the doctor’s note. … The parents have a unique perspective on their child’s well-being that, as medical providers, sometimes we’re just not in tune with. Because we don’t see them every single day. So I think the parents keeping track of it somehow is super important”.
Parent expectations	“In the NICU, at least for us, we weren’t allowed to do a whole lot. … When we were finally able to go home, I didn’t know what to do because I was never allowed to do anything”. “One of the most traumatic parts of our time before coming home was the transition, we went upstairs [pediatric ward] for the last like 8 or 10 days that we were there, and that transition happened really, really fast, and it is such a different world up there. In a lot of ways I feel like the transition upstairs was harder than the transition home because we just, suddenly nurses were like okay, so you’re gonna give him his medication now, and we were like, oh, okay, we can, but we’ve never done this before, so we will need a little instruction now, and figuring out the expectations, in the NICU, where there are things you’re not allowed to do versus upstairs where there are things there that you’re required to do”.	
Uncertainty with durable medical equipment (DME) and ongoing needs at home	“Not having anybody to talk to or call, in case something went wrong, in case my baby was sick, and then in case I had any other questions, or I forgot something. I wanted to have somebody that I could talk to and ask. And that was scary because there’s a lot to learn. There’s a lot to know when you leave that hospital. I mean you’re doing the job of the respiratory therapist, two nurses, the everything. And you have to really know your baby and be able to recognize signs if something’s wrong. “Once you go home, you’re on your own. And that’s really scary. And when you call back, they’re like he’s not our patient anymore, call your pediatrician. Well my baby hasn’t seen the pediatrician yet. So what about the time between your discharge and see[ing] your pediatrician? And I’m not even all that confident in my pediatrician, (a) because they’ve never even seen my baby, they don’t know his special case, and he has quite a complicated history, and (b) because he’s basic. I’ve been dealing with specialists this whole time who are really in the trenches with my baby…” “They give you a printout to care for your baby at home. But the truth is it’s kind of a guessing game. For example, my daughter, she’s on oxygen, so, she has the nasal canula and she has stuff taped on her face, and no one ever taught us how to take it off, or put it back on, or how long could it be off during changing or do we take it off during bath time? No one gave us these answers or even the opportunity to ask these things. When we got home, we were guessing, trying to reach out to other families that had children on oxygen at home to get those answers”. You have to know the equipment. It’s a lot. The person comes and for 15 min [and] reviews the equipment with you. But when you get home and there’s like seven tubes, you’re like oh gosh, just for one machine, you’re like what does this one go to? … So maybe when you leave, if there’s like a video or something about these questions that you could watch … And they have to all be changed at different times. …It’s a lot. It would be cool if they have maybe a binder for you that had easy things, or just general information that’s like everything that you needed to know where you went home, and then it’s like a number that you could call somebody 24 h a day. Because like my pediatrician, he’s only available weekdays Monday through Friday, which to me sucks, ‘cause my baby is super at risk for stuff, and he has a very delicate. And the last thing I wanted to do was, I really wouldn’t have anybody to call for basic stuff, like what did you say about doing this, or you know what I mean. And it’s like the last thing I wanted to do was take my kid to a urgent care, an emergency and sit in there with all the other sick kids, you know. Because what might make another kid sick could kill my baby. So you know, scary, it’s very scary. And you’re coming from the NICU, you know what I mean. Not like it was just regular hospital. Like this baby was in the NICU for months”. “I was very anxious about his care since I had never taken care of a baby with his condition before. I was anxious and nervous to be doing it on my own, and not have a nurse there to talk to. One thing that would be helpful is the first month or three months or something, to have [an] emergency number or a contact number that you could call specifically about your child”.	“If I have to identify one thing, as simple as it is, I think that with our patients, they’re so complex after leaving the NICU, that it’s overwhelming to try to get in touch with all of the clinics. And something as simple as not knowing the clinic information is a huge obstacle for them. So, I really liked that there was a link, or a folder, that had the [hospital] directory. I know it’s pretty simple and straightforward, but that’s really a huge benefit for these families, to have everything readily there at one touch”.

**Table 4:** Themes and illustrative quotations, impact of uncertainty.

	Patient	Provider
Impact of Uncertainty		
Emotional: guilt, blame, isolation	“I just felt very disappointed in myself. I just felt like I couldn’t do anything well, and I couldn’t do anything right, and that I wasn’t the mother who I imagined myself to be, and I just wasn’t the partner, I was failing at everything”. “They used to tell me he was doing good, he was a healthy baby, but I was just blocked in that part…I started blaming myself, probably I put him through this for waiting this long to have a vaginal [delivery]. …Then the doctors keep explaining it’s not your fault. If we knew this was gonna happen, then we should have just told you go right to C-section. So I was just blaming myself for everything that was going on, that I wouldn’t let nobody tell me this is correct, this is the right thing”. “There are lots of triggers. And I had to deal with my own feelings of resentment and jealousy of people having normal pregnancies and normal deliveries, which doesn’t feel good. It feels really awful to not feel like I am, I want to be happy for my friends, and I don’t want anybody to have the same experience as I did, and also it comes back to this like why did I deserve to have this thing, and it’s just this very weird space emotionally and mentally”. “I couldn’t concentrate at work, but work was kind of the only thing that I was still able to do okay at, because I didn’t talk about anything there. So nobody even knew what was going on. And not having maternity leave, like I was pregnant, and then I wasn’t, but nobody knew. And it was just very weird, and I just kind of isolated myself”.	
Social networks	“Our families really didn’t know how to support us. My mother, she’s a social worker, and she’s someone who I really was counting on to be strong and to know what we needed. And she could not handle any of it. She was here when [Baby L] passed away and it really broke her. I understand that, but at the same time, it was like I can’t have you in my space and have to take care of you. She did not understand that. And that has been really hard on our relationship”. “I didn’t have any support at work, and I really lost a lot of support in my friend circle, just because people would say things like well at least you have [Baby A] or just weird [stuff] like God always has a plan. To me, those things are just not comforting and it feels very dismissive of the reality of the situation, and the gravity of the situation”. “I think when you lose a child, there is nobody that you can, like, I don’t know. It just was very isolating. I think even just having a NICU stay, where your child, like that’s traumatic in itself, but I think when we lost [Baby L], it just put us in a different realm. And people don’t know how to talk to you and nobody really knows how to support you”. “I think there were papers that were given to us, like you can go to this support group. But I didn’t feel like those support groups necessarily dealt with what we were dealing with, which was we have a newborn and we lost a child. So we have a loss and we have a celebration simultaneously. And that situation is very unique in itself. I didn’t feel like we could share the space. And I’ve tried different support groups, with the loss of a child. But it’s not the same, because I do end up with like survivor’s guilt because we have [Baby A]”. “I mean, COVID, we knew we were kind of keep[ing] him under wraps ‘cause he came home in January, so even in those 2 months we were very choosy about what friends we saw. Now, we don’t have family out here, we’re not seeing anyone and so all of those mommy bloggers who are like, have someone come over and take the baby while you shower, like that’s just not in our toolbox”. “I would say trying to manage working from home and some of unemployment as well as trying to care and he needs a lot more attention than a typical kid would. You know he has seizures, so we have to really be watching him all the time, so I’d say all of that kind of under the umbrella of like not being able to have babysitters”.	“One of the things that happens is you get home, and you have 20, 30 messages on your answering machine for people calling, wanting to know how you’re doing and what’s happening with your baby. So one of the things that I always suggest is to have an outgoing message, basically updating people, giving the information that you are comfortable giving for an update. And letting people get that so they don’t constantly try to reach you on your cell phone or on your home phone to find out what’s happening with the baby”. “That was a way for parents of NICU kids to give out a website and on that website, if they were given permission by the parents, they could look at the information as to how the child is doing and what’s happening in their lives. And it’s one factor that a lot of people can go to so that they don’t have to constantly call the parents you know. Then the parents have to repeat the same thing over and over. Something like that was very helpful to those parents and I think it would be helpful to NICU parents”.
Parent–Sibling relationship	“I think it was really tough on my relationship with my stepson, because we’re very close, and he didn’t understand why I was away all of the time, and why I was sad. He was very young. [Boy E] was only two when the girls were born. It was just, it’s a lot of stuff going on”.	“Every parent knows their child best and knows, what they can manage emotionally and psychologically in terms of exposure to what they’re going to see in the NICU…For some kids it’s fine to come in and actually be exposed to everything going on. But for some kids it’s really traumatizing. And so [Skype or Facetime] is another way of having a connection with the sibling that’s in the NICU and yet not having to see other babies and other machines. And also perhaps not having to see certain things that they don’t even necessarily have to see on the child themselves. They might see their face and they don’t have to see different things that are attached to their bodies and coming out of different places. So I think that that would be good. “I think the general rule with siblings, parents should always pay attention to how they’re eating, sleeping and their academic functioning. Because those are the red flags that go up. But they should be aware that siblings sometimes feel guilt and responsibility for bad things that happen. “If the parents notice acting out behavior, which also comes from a lack of attention. The kids are suddenly kind of thrust aside, a lot of times given to the care of other people. And then problematic behaviors can start, developing there because they’re not getting the attention that they need. I think that parents should be aware of that and you know that quality is more important than quantity. In other words, spending time with each child individually and talking, letting them talk about themselves, not necessarily about the baby, unless they want to talk about. You know but really focused attention, even if it’s for short periods of time it can do wonders”.

**Table 5:** Themes and illustrative quotations, barriers, continuity of care.

	Patient	Provider
Systemic Barriers		
Insurance	“I had the biggest issue trying to get her prescriptions because of the insurance. I guess there was an insurance issue with one of the medicines. Like it had to be preauthorized or something. When I did get discharged, I went down to the Walgreens and they wouldn’t give it to me…So I went home and then I came back with the card, and to find out that I still couldn’t get it because it was on some type of plan program whatever and it had to be pre-authorized by the doctor or something. It was just a whole complicated mess that upset me ‘cause that was a waste of time, twice”.	
Appointments	“Scheduling is the hardest thing, because I know that everything is so backed up all the time. The high-risk infant clinic took us months and months to get in. … And then just the referrals for insurance, it takes a while. And it’s very frustrating because you think like okay this is so important [for] my son, you know your kid has to go to this. But you want me to spend months and months waiting for an appointment”. “Which programs? All of ‘em. But not right now because of the coronavirus. So his physical development therapy has stopped. They cancelled my full STEM. I’ve been having a hard time contacting them, or I’ll contact them and be on hold for so long. Then my phone dies or they hang up or the worker’s not signing documents approving the full STEM for a certain reason”. “The most challenging thing was transportation, driving to LA”.	
Continuity of clinical support, inpatient and outpatient (positive and negative)		
Continuity of Clinical Support—Inpatient	Her primary nurse was amazing. As we got closer, she was like I don’t want you looking at that. Like you are not gonna have this when you go home, and you need to get comfortable with reading the signs from [Baby A]. She would set up the space so that I couldn’t see the monitor, like it would be behind me. I do think I have trauma from the noises, but it was very helpful. Her team was so amazing, but especially her nurse and nurse practitioners, they gave me the time, they gave [Man D] and I the time and patience. We had a lot of questions, and they really met us where we were, and talked to us candidly and honestly about what to expect, and some of the things that parents are worried about to help us just feel like we weren’t the only ones, and how they would deal with it. And we took that to heart. That was really helpful”. “The nurses were fantastic. I spent a lot of time with them. I actually had primary nurses, and I think that that was really helpful because they really got to know [child M.] and I really got to know them. Although we did have some times where we had a different staff members watch her, having that consistency, I think was really beneficial for myself and for the baby. And probably for the staff too, because they felt comfortable and they knew her. If she’s having an off day, or something, because they were able to pick up on those things, it meant that I was going to be able to, too. So, me being consistently with her, I was gonna learn stuff about her and that would help make everything more smooth”. “having a new resident every time was very frustrating, because it was like you don’t know my baby. And if you’re making these decisions, I’d appreciate that we have a conversation about this, so I can catch you up”.	“When you’re coming from NICU to outpatient, which is a whole new world in terms of how things are set up … So in terms of a place to add, I think nurse case managers are an important part of social work. … And I’m saying nurse case managers in terms of supplies, medications, getting authorizations for those specialists or those supplies or those medications. That whole thing is a nightmare for a lot of parents, that coordination piece. All of a sudden, they’re like you’re discharged, here, figure out this whole crazy situation”.
Continuity of Clinical Care—Outpatient	“There’s one person I can call at [the clinic] and he lets me know all of the things that are happening. If I were to call him and I forgot all of the appointments, he’ll run down through all of the appointments, all of the notes the doctors have talked about, [and] detail his condition. He’s a person I can go to to really get all of this information, and I think that’s been very useful. “There’s a lady I call from [the hospital], but she works with both parents. If I feel like I need something, I’ll call her…And I was telling her the other day, I always have a hard time when he’s eating and I have to go out somewhere, because I have to find a way to prop his machine, prop his bag and everything. So they’re like you didn’t get a backpack? I was like no what are you talking about?…15 days later, I got a backpack in the mail from [the vendor]. I was like oh my god, this is so cute. I’ve never seen it. It fits the machine perfectly and then it has the plastic, so even without unzipping it, you can still see the machine, like the buttons if you need to adjust something. And then it has on the other half of the backpack, you hang a little feeding bag on there”. “The regional center has definitely been one of the programs I use the most, also WIC because that’s how we get his milk, so we can put it through his G-tube”. One of the programs that I forgot to mention that I think has been very helpful is the California Children Services, CCS, which I only learned because of being there at the hospital. I think that that’s one of the most helpful ones just because it covers whatever Medi-Cal doesn’t cover for him. That’s one of the reasons why he’s able to have his hearing aid and access the equipment that he needs to make sure that he’s able to thrive, so, like his oxygen”. “Because before, it was like I cannot afford it. I’m gonna have to borrow money for gas. The social worker told me about the insurance helping as well. So that was the biggest help”. “[Baby A] has ongoing PT virtually through the regional center. And then she has infant STEM through the regional center. The regional center’s amazing. Being linked in with them has allowed us to reach out if there’s any concerns. They also reach out to us. There’s all of these levels of testing and evaluations and letting us measure where she’s at. That’s very informative. Then if there’s anything else that we need, the regional center helps us to get those things for [Baby A]. I feel like we’re pretty well covered”. “At the NICU, they had helped us to make first contact with the regional center for any of those supports. That was really helpful. And we followed up on that. I felt like we did a very good job of trying to understand what we were dealing with, and making sure that we were following up, because you have to be able to do that. I don’t know if I feel like we were very lucky to have the team of people who could talk to us about this stuff, and tell us that these are the things that are available to you, and you should take advantage of them…I hope that information is available to everyone. I think it’s very important. For the NICU to make that first contact with the regional center for us was really important, ‘cause it’s hard to do that”. “There’s a lot of appointments and a great team of folks that talk to each other. When I go from one appointment to the other, they already know what one or the other has been talking about, [which] has been very helpful. He’s gonna have another surgery, so he needed to get cleared by all these specialists, but they were already talking to each other before I even got to the appointment. It makes me confident [in] the work that they’re doing, and it keeps me at ease knowing that he’s in good hands”. “I think it’s a little easier, because it’s virtual. Sometimes [child M.] will be napping and I’ll just text and say hey, you know, is it okay to kind of move it to a different time? And for the most part, it’s been fine…I think rescheduling is not very burdensome, and I think that that’s something that is helpful. It being virtual allows that”. “Thanks to the staff at [the hospital] that signed him up for the school district very much involved in his language development, and have been giving me a lot of resources of apps, and books, and even a parent group we’ve joined where people have different children with different [conditions]…Trying to navigate this space has been possible because of the resources that we were given before leaving”.	“Each clinic should have like a group email for the dieticians, or nurse case managers, or social workers. If you are stuck on one of those pieces, like you’re stuck on formula, or G-tube supplies, or mental health, you could theoretically shoot an email to, or make a phone call to one of those groups to help you with getting supplies, figuring out the insurance. I’m bringing that up because that is routinely often what the big issues that we’re seeing when people come out of the NICU into outpatient world, is how do I coordinate all these supplies, all this medication”. “I think talking about the transition from the NICU to outpatient, which you have this framework of support, social work doctors, and then you’re going to outpatient. You no longer have your NICU social worker and you have maybe five clinics that you’re going to see in the outpatient. Who is your point person for psychosocial stuff in that it’s kind of confusing, and a shot in the dark. If we could help them in that way, in that interim period to access a person to talk to if you have more questions”. “The dynamics change slightly. It’s a little less nuanced online. You can’t touch and hold someone’s baby. Your baby can’t move around and interact with other babies. It definitely changes how we socialize. That is a missing important piece of how we connect as humans when you’re doing it online. I’ve found all the Telehealth work I’m doing right now that I wasn’t doing before, that it is really, we’ve been able to establish rapport, and make connections, and support people and do the bulk of the work at the same quality”.
Continuity of care, outpatient—Challenges	“I feel like the pediatrician is a very basic level of care, and not familiar with your baby. So I really just don’t have that much confidence, even now, in my pediatrician”. “Whether it be a nurse’s follow-up or a social services follow-up about how are things going. Like it’s been two weeks now, are you doing okay, everything going okay? And at that point, going like, would you like us to call again or are you good now. I think that would have been helpful in this scenario”. “For the family, in the hospital, you get an immense and a wonderful amount of support. There [are] a couple people that would go around and talk to you and stuff, and that was a really great thing to have in the NICU. There’s not much resource outside of that, though. I almost feel like that [should] be continued. For the following month, something like that. Or at least someone to call and speak to about certain things”. “I definitely think having the resource to reach out to nurses or physicians that would get back to you quickly, and for nonurgent things. How do I make sure this dressing is correctly applied? Things like that. ‘Cause if you reach out to your doctor, you know that could take several days for them to get back to you”.	

**Table 6:** Themes and illustrative quotations, coping, witnessing progress, and adaptation.

	Patient	Provider
Coping		
Information Gathering—parent empowerment/coping efficacy through information gathering	“I Google stuff, I ask the doctors when I go over there. Like when I took him for his pediatric surgery evaluation, I told her I know how to change tube, but I want you to walk me through it. She goes how about you show me, and if you have questions, or you hesitate, I’ll step in. And I was like okay fine. And she goes no you have it down. I’ll ask the doctors, I’ll use the app a lot, I even Google stuff if I need to know something. I’ll read the parent magazines. I’ll use that one a lot too. And then if anything, I’ll ask my mom”. “Even [when] the nurses were there, I was doing baths. I was always watching. I knew what time and everything. Two weeks before I left the hospital they let me do it by myself. They just went and watched me and [brought] me medicine. That was helpful because when I’m [here] at home, nobody else [is] watching me so I got to learn how to do it”. “The G tube class that they have there, I took that and I was already confident about it because I knew she was gonna get it. So I did my own research on it. [I] already knew how to connect it and change it and stuff. So, I was pretty confident when I left the hospital”. “I asked a lot of questions the whole time that he was in the hospital. Honestly, that’s where I got the bulk of my information. And when I go back, I still ask more questions, whether it’s the nurse, or the specialist, or the doctor, or the pediatrician. I’m kind of a question [asking person]. I really haven’t done much online besides look up the actual disease that he has, and just to re-verify what exactly it means, how you get it, what the prognosis is for it, how often it occurs, that kinda thing… it basically was reinforcing what I already knew”. “The doctors explain[ed] things, [gave] me the signs and symptoms, what things to look for, and the training they gave me with the CPR, and gave me the doll, the kit to give it to my family, so they can work on it and watch the signs and symptoms. That’s what made me more confident about coming home. The training”. “We had some great nurses. We learned a lot from them. We didn’t really take classes, this is our first child. Our family wasn’t really in town yet, so we would’ve had to do things all on our own, which we would’ve managed. We were competent, but at the same time, it’s like, after dealing with the nurses there and their expertise, I felt very comfortable”. “The nurse that was there on discharge day, she was really helpful. She showed us how to give her the medication. She wrote out timetable on when to give it, how much to give. That was really the only thing. She had like three medications. So she documented well for us how to do it, and when to do it, how much”. “The high-risk clinic I think has been very helpful in me understanding my son’s development, so we can assess where he is at with the test, and understand even though he’s eight months in age, he’s, for example, his fine motor skills are at six months, his language skills are at four months. For me to be able to understand all of that and get all of that information I think was very helpful”. “When I was in the NICU, I did receive a CPR kit, but we didn’t do a class or anything to show me how to work that properly. I had to watch a video myself. A class in that area would have been a great thing”. “All of the people that were there were really supportive and very engaged in ensuring our success and providing a lot of support, not just with resources, but also with the openness saying you have any questions, please feel free to ask them. And when we asked, they were answered”. “Even coming home, I still have the support because even when I go to his appointments, I can always ask these questions over and over again, and they’ll always answer these questions for me in a way that I understand and give me all the information I need”. “The whole experience was stressful, but it was very much so helpful where I got all the information I needed to handle everything that I needed to once I wasn’t there anymore”. “I mean you have to push this thing into this mouth. You’re scared of hurting your baby. I got it down now, but at first, I didn’t even want to touch it. … Even to address those concerns, you have to be very delicate, it’s okay to go slow, take your time. And as you do it more and more, you’ll become more comfortable, and it’s not as scary, not as hard. There is a whole anxiety that goes around that G-tube. Like I cried about it. I was wondering if I was making the right decisions to let ‘em, you know. … It’s just very scary”.	“We teach them these things in the NICU but if you don’t remember, you can just go back to the video and see how things can be done”. “Being a bedside nurse, I would hear it from the doctor, what they said. And you can see them nodding and nodding. And then, afterwards when they leave, and I’ll say, “So did you understand what the doctor said?” They’re like, “I think, but I had a question”. I was like, “Oh, my gosh. Why didn’t you say anything,” because they feel they’re just bombarding the doctors, and like their time isn’t worth it”. “I think we do a bad job in general in the NICU. Everyone’s always does mom know, did anyone call mom, did we talk to mom. I feel like the dads are so often left out. I’d like to see like more stuff for them, more ways that they can get involved and be helpful, because I think a lot of times they do kinda feel like I’m not the important one here. It’s like no you are”.
Parental mental health: - Ongoing process - Provider: importance of parent self-care	My breakdown that I had, like I thought about leaving my family. I thought that they would be better off without me. Things weren’t getting better for me, and that was something that people weren’t picking up on. You know how after your baby comes home, the first couple months are challenging, but then it gets easier. For me, it wasn’t getting easier, it was getting harder, and I was getting more anxious, and I just wasn’t getting better. It was getting harder, it was more challenging. I was more on edge. And those things aren’t normal. And I feel like every time I went in for these things, I would try to express that. And it wasn’t acknowledged and it wasn’t met with any services. “I do think that having more specialized care and understanding maternal mental health is something that is just a huge gap, and I think that it’s really important that we do a better job with that. …. And I think that we really need to look at it as if it goes untreated, you’re dealing with that forever until it is treated. It doesn’t just go away. But also we really need to think about it more like three years post-partum. It’s a much longer trajectory I think you’re dealing with things, and they manifest in different ways”. “The best part about it was the nurses. We always joked about it, but I loved that they always pushed to go outside and take some time for yourself, like we got this, go outside and get something to eat, get some fresh air. I had that constant update and reminder all the time from all the nurses, like take some time for yourself. And I appreciated that”. “I know the social worker really encouraged me to listen to my husband to go home and get a couple hours’ sleep, take a shower…the nurses and doctors encouraged us to stay positive and told us that she’s doing well so that mentally calmed us down. Like, calmed our brains down to all the craziness”. “At the beginning, it was pretty scary. I didn’t know that the baby was coming with such difficult conditions. So it was very helpful to have the social worker there explaining what was happening, but also reassuring that there’s always support and help for myself and the baby”. “Talking to the psychologist that they had was helpful for me to have peace of mind that what I’m doing and what I’m thinking are the right things to really support him”. I’m glad I told [Man D] because I was just like, I went to work, and I was like they will be better off without me. And it wasn’t suicidal. I wasn’t like I need to die, but it was like I need to leave them, because I am causing all of this. Like what I bring into the household, what I bring into this equation is taxing on everybody, and it’s not healthy, and I don’t want my kids to be around it, and I don’t want [Man D] to have to deal with it, and he’ll be better off without me, and they will too. And that is not normal, and that’s not a good thing. And I’m glad I told [Man D] and didn’t just like leave. “I didn’t feel supported through the follow-up appointments. I didn’t feel supported from the hospital necessarily. I had all these meetings with all of these people about everything, but it was never [enough]…‘cause what I’m dealing with, and still dealing with, is some maternal mental health issues, and PTSD”. When I told [Man D], he was like okay this is definitely more serious than I imagined. He immediately got me an appointment with somebody. … And thank goodness, the first woman who I got my appointment with, she turned out to be really wonderful…she did end up recommending that I get a psychiatrist so that we really figure out what’s going on. Then I was diagnosed with severe PTSD, and mild anxiety. … And I’m a work in progress. And we’re just in that space. I do think that I’m in a much better place now”.	“Parents a lot of times don’t care about themselves when this is going on. And they let themselves go and they get sick and they get stressed out. And they’re not paying attention to themselves … But I think when it comes to knowing that how they are will impact their children and especially then they’re more likely to be monitoring and taking care of themselves. Because now they know it’s not just about them. I don’t care if I’m get any sleep and I don’t care if I’m not eating. But when you realize that if I don’t do that this is not good for my baby, that’s where people kick in and really make sure they’re more on top of things. There’s lots of studies on social referencing…when you have a toddler that starts to walk and that toddler falls down, the first thing they do is turn around and look at the parent’s expression. And, if a parent is in shock or upset, that child immediately starts crying and screaming. If the parent is like you’re okay, get up and go, they get up and go. And so they determine how they’re doing by referencing their parent’s facial expressions. I think if people knew that your baby can, can sense how you’re doing and that’s why it’s so important to keep yourself as healthy as you can be and well rested. Because this does have an impact on your child because they sense these things you know”. “Conveying to parents that you, at some point you need to take care of yourself alone because you should be healthy and maybe you have other kids and just to get through this you need to be healthy and strong. But it’s also important because it does impact the child”. In the NICU, every parent gets screened, if they’re showing symptoms or not, every parent, every week. Based on how they scored on that, then they would have an order to have someone from the psychiatry department come and see them. I hope they continue that once they leave the hospital. I don’t know how much follow-up [there is] afterwards.
Parent–Child Connection	“One really helpful component for us was music therapy. We’re both really musical and we couldn’t hold him for the first month and that [was] hard. I think we kind of built that bond through music …We had so many people looking after the medical side of things, but that really helped us build that connection to him when we couldn’t especially when we couldn’t cuddle him or after we finally could hold him, it was still quite a process to get a baby on a ventilator into your arms. So I think that really helped us like learn how to connect with him”. “Another thing that my wife really liked, is the fact that the library is always giving a book every day to the children and babies. Or for parents, they would play music, or the pets would stop by. Those things really added a lot. Because that’s why we actually look back and we have good memories of CHLA in general. So those things help”.	
Social Support	“We had the visitor from the chaplain, she would come by and was just so kind, thoughtful, and genuinely caring. She would just come in and check in on [Baby A], and she would check in on [Baby L] and [Baby A] when they were together…She was a really wonderful presence in the hospital. I could tell that she cared, and she offered her services, but it didn’t come across as pity. And that was always a hard thing to look and see in somebody’s face that you’re dealing with. It was just like we don’t need your pity”. “We [had] such an amazing community around us. They held fundraisers for us while we were in the NICU and people were like, we will come babysit any time. I know we can eventually cash in those favors. We have a lot of that emotional support, but not being able to have any of that, that’s hard”. “We had a really good friend of mine set up a Go Fund Me for our family because [Man D] wasn’t able to work, and I was working. [We had] just one income for our family and [were] dealing with the hospital and everything. So she set up a Go Fund Me for us, and that was very successful. People banded together and helped our family in that way. That really helped us to be able to cover rent”. “I had another girlfriend of mine come out, who was really supportive, but she works in hospice care. She was equipped to help us. She just came in and didn’t need anything from us, and made us food, and bought us dinner. She basically fed us and helped me organize my breast milk. We got in the freezer and she dated everything, and she helped streamline things and just made things really easy while we were going to the NICU”. “Having my husband around and then having my best friend here just to help. She’s been through 3 kids so it’s been helpful for her to be here and then to have her kids here just to keep my older daughter distracted. Because I did feel kind of guilty about having that time away from my older daughter. And that played into the emotions I had but I was watching myself to make sure I didn’t get into any postpartum depression or anything like that. So, I think just having people around and being able to share my feelings at that time really helped”. “My father was a godsend, which is so strange, ‘cause he is not that [person], like he’s almost like oblivious. But when he came out here, he was self-sufficient, and did everything that he could to help us. He made us food, he took care of [Boy E]. He would take [Boy E] on walks. He would get up with [Boy E] in the morning, ‘cause we would go to the NICU at three in the morning or five in the morning…And then we would come home. And my dad was just so amazing with everything. And that was something that was totally unexpected”. “We have a lot of family help, especially my mother. She comes for about half the week. And at the time, she was with us pretty much 24/7 for five days a week. It’s hard enough with one medically complex kid. Then adding a three year old at the time just made it a little bit more crazy. She decided to stay with us until we got our feet up from under us. We were in such a like mode of action for so long, with the emergency delivery, and the NICU, and just everything, and making everything work the way that we needed it to work that [Man D] and I are a high-functioning team, and we figured it out. And we did a really good job. But I think that my mental health is, has, was, is still probably the largest struggle for us”.	
Social Media	“It was hard for me to find a group that held my same values…we’re very pro-vaccinations, pro-science research and doctors, and I found a lot of groups were not in that same direction. But I found this one group that I really like, and they are all sort of on that same, wavelength and that that’s been really helpful”. “Through Facebook, I’ve been connecting to the groups that I’ve been talking about—parents that have children that are hard of hearing or deaf. That’s where I’ve been getting a lot of parents’ feedback on what are the things that are working and not working, which has been great. Through Instagram, it’s mostly posting pictures of the baby. And really, for me, I think it’s normalizing the fact that this is our new reality of a child with special needs. I think that for me, that’s a way of showing people that he’s no different from my daughter or any other child”. “Sometimes with professionals, it’s like yes I appreciate the knowledge you have, but it’s not the experience. And on the webpages, most of them are parents. So they’ve been through it the same. They’ve been through the same things, or they’re going through the same things. They’re like oh tips and tricks, like this might help the baby, or this might soothe your baby, stuff like that. “Even on Instagram, I have started to follow a couple of women who have G-tube babies or oxygen babies. I rarely ever comment, but it’s just good to know and to see that there’s other people who have babies with similar conditions. Some of ‘em have oxygen and G-tubes, and they have other conditions and they just happen to need that oxygen… It is comforting for that.	“I think it would be highly valuable. What happens when they’re inpatient is that they are encouraged to participate…[the social worker] ends up almost encouraging the mom to feel like, “You need to find an outlet because your baby has a special diagnosis that a lot of babies don’t have. So it’s better for you to go and find a support group”. And then, what she always says is, “Go find one on Facebook”. I don’t know how reliable Facebook is, in terms of support groups, but I know that they just need an outlet. So that’s good. “I loved how the parents were connecting and supporting each other. I loved how the facilitator promoted that. I felt like a lot of the information that I give families in the high-risk infant follow-up clinic, it was kinda nice to be able to give it all at once in a way and have them be able to process it in a more meaningful way, because it was in that setting with other parents. So I thought it was great, and I definitely think more parents would welcome something like that and ask for it. A frequent question we get in high-risk infant follow-up clinic, is about connecting to other parents. So I think it’s wonderful”.
Other NICU parents	It’s parents with experience. They’re going through the same thing that happened with you, not a professional who’s only dealt with it by reading…they know what they know, and they’ve put their time into knowing and trying to help others. But I guess it’s different having the knowledge, and having lived experience”. “Through friends of friends, people who had lost children, reached out through our friends, and were like I’m here as a support. If [SELF]’s comfortable, you can share my number, or I will reach out to her. That has been really helpful. “The woman who set up the Go Fund Me [for us], she had a stillbirth and she was one of the main supports for me, because she was a bit out from that, a couple years, which was enough space for her to be able to talk with me about it without it being overly painful for her or too close. We were able to talk very honestly about just everything. And that was just really helpful, and just knowing that there were people who were available”. “I think that is valuable and it kind of goes back to the whole a parent that has experienced similar things. And I love it. I think it’s very valuable. Even with the support group, I don’t feel like we compare our kids at all. Especially because they’re so different and they’re even at all different stages…I feel like it’s very much like oh, we’ve been through that. I could be the one that has the youngest kid now and then somebody comes in who has a kid that’s younger. And then I’m able to pass that on. And that’s what I love, is I can get from other people and then I can give”. “In the NICU, they had different activities. They had the day where people just go and have coffee or tea. And you were bonding with other parents. They also had some type of arts and culture, where you paint and things like that. And then they also have this really great program where they bring you books to the room and you’re able to read to your child. I think that what I was talking about is like the human aspect of things, like having other people and sharing in conversation what’s going on. I think it’s always very helpful when folks have similar situations, like having your kid in the NICU for whatever reason, or whatever condition. It’s just knowing that there are people there that also are going through the same experiences, and even I guess like reaffirming that the services that we were receiving were really good. I think that that’s one of the things that, at least for me, was very helpful. When I was there, it was like two days into the process that my kid had just gone to the hospital. I think there was a family member just told me look, my kid’s been here for three weeks, and they’re great, they’re treating him well, and they’re making the best that they can. Hearing those things and [reassurance] from other parents, I think that that’s always good from the experience of other people. So it made me feel more comfortable that I was making the right decisions of where my kid was being treated”. “When you are with another kid in the room, there’s also always that support that you can have from parent to parent understanding each other and knowing that you’re not necessarily alone in this process, that there are other people that are going through similar situations. One of the things that I think for me cannot be substituted, it’s this human contact of actually talking to other people”. But through the maternal mental health world, there were some support groups that came up, and they were discussions, and holding space, and doing a candlelight [vigil], like a circle of mothers who would tell the stories and hold space for their lost children. I joined that, and it was amazing. It was four hours long. But it was an opportunity to share your story, and to talk about where you are, and it was mothers who had lost a child maybe 11 years ago to a mother who had just lost a child. It was a range. I tried it, and I thought okay this is a good thing. And sadly the comfort that we have found has been with other people who have lost children. It’s a weird space to be in because those aren’t our friends necessarily and I just think that again, we made the social worker at the hospital uncomfortable, we just make people uncomfortable, because it isn’t something that we move on from. It is, now it’s just part of who we are.	“The other social worker would actually sometimes, this is pre-COVID, would find a different mommy who had already ridden the ride on this bike, who was already discharged… She would almost be a sponsor… I feel like whenever parents have some support in the way of having a group for their diagnosis, it’s a lot better than just having a support system, where it’s like, “Oh, yeah. You do have an intact family. You do have like a good religion, a good household”. But then, sometimes it’s really lonely walking that path by yourself if you didn’t know anyone with it”. “The NICU’s really overwhelming. So if they’re going through something specifically and then you find somebody else that is going through that as well, I feel like you, the parent might potentially feel that they can talk to somebody…so I think very useful. Most of the parents sometimes feel like they’re very alone in the NICU and they don’t have anybody to talk to, especially if they don’t have support within their family. So yes, I think those friends are usually useful”. “Because the hospital is moving towards a patient experience type of strategic plan, I think it would be really great to partner with a peer as well. So, somebody that has been around the block. There are these groups called PFAC, patient family advisory committees, that have ones in Spanish, ones in English, and different advisory boards that have been created that can actually be leveraged to help cofacilitate this with the social worker, and I think that’s a lot more powerful when you do have somebody that does that”. “I trained in a NICU that was half individual rooms and half a big open room with a bunch of babies. We closed that space in the middle of my training and moved them in there. The parents that came from there felt [they] had like a little family and a little community in that big open style room, and now [they] feel really isolated. I know parents will meet up on their own in the lounges, but especially now with COVID, we’ve limited so much of that. I think that anytime NICU parents can connect, it seems to be very helpful for them”.
Spirituality	“I feel for me God was everything. He was the one who give me the strength. I feel God has give me my daughter. He’s the one who’s going to take care of her. I try to be the best positive, trusting God that everything was going to be okay. One day, if that was God’s decision I would bring my daughter home. Otherwise, I would keep my faith if even my daughter will not be with me…I trust God and whatever decision he will make is the best, even though it might be painful at the beginning for me. But he will give to me the strength. So everything I focus on that for me. God was everything. Right now, I feel so happy and joy in every stage of my daughter”.	
Adaptation		
	“I really felt confident that she would do better just being out of the hospital. It was so bright in there. It just wasn’t a natural place to get good rest, and I never felt like she was really resting well. I felt like she rested well when she was with us, but I just felt like she was so exhausted. It’s exhausting to be in a space like that. It’s loud, there’s so much going on…There’s just so much lost time of talking and interacting and human contact. And we don’t know what that really means for her development, and we’ll find out. I just was feeling very confident that when she came home, that she would thrive, like being home is what she needed to do”.	
	[Social media support] is amazing. It became one of my largest avenues of support in some ways, because I didn’t have to put myself out there, but I could learn so much. I could use my knowledge, and my strengths to figure out what it is that I can do in that space. That was very empowering I think especially when my self-confidence and the devastation of loss, and feeling very helpless, it made me second guess my abilities, and my strengths, and just everything about me. And then finding these spaces and being like no, I know this, and you know, I’ve also had these experiences, and I’m also in a position to further this work, was very reaffirming for me. That has been very helpful in this journey of kind of picking up the pieces and feeling more healthy and capable”.	
	I was confident that I was able to do it. I honestly believe you guys train really well…the transition was really well done. I felt confident that I’m able to do [it]. And the team was very resourceful.	
	“My baby had open chest surgery. He has a G-tube. One of the successes that I feel are huge for us is our family being able to understand, especially being able to explain this to our five-year-old, that her brother is different in many ways. I think that has been helpful. The child life counselors at the hospital gave my daughter a doll with a G-tube on it. So I think that that was something that we were able to bond around my kids’ difference, just because my daughter, while we feed her brother, she also feeds her doll, in a different way, like not with a bottle, like we used to. So I think that was something that has been able to transfer from the hospital to the home”.	
Supporting others	I’ve even thought about doing a page for him, or a blog for him, just about the whole journey, because it’s scary. Even when I see their posts, it makes me cry, ‘cause it’s like I feel sorry for them, just like I felt sorry for myself, and my baby. You wish that there was something that you could do, and you know a lot of the pain that they’re going through. I like to think that it is a journey for us, and it’s his journey and my journey. And then at some point, I would like to think that we’ll be able to look back at it. It would be helpful to other women so that you know you’re not alone, ‘cause it is scary. I do have my moments. But I can see where that would be helpful for a lot of women”. “Getting connected with women and hearing stories helped me to realize that I was very fortunate in my space, but that there are all levels of care and access to care. That was really helpful to understand that we’re all kind of dealing with these traumas, and what does that mean, and it gives me something to focus on. I don’t feel hopeless, because I feel like there are things that we can do to change these systems and make sure that this isn’t the way that people are left feeling. If you’re getting into mental health and just general care, when you’re dealing with moms who have had trauma and who are being re-traumatized through every level of care following, it’s like okay so what does that mean for our families, and for our children, and for everything else, and for the world, and our work space, and how we’re interacting with everything. It’s just been really good, and I think it has put me in a place of feeling like I’m not alone in noticing these issues, and that they’re very important. And that to me is very inspirational, and it just helps me to kind of feel empowered to do something, or inspired to do something”.	
Experience of Uncertainty—Adaptation through witnessing progress		
	“That bond between us and making him know that he’s loved and supported, and seeing him emotionally develop, because he has global developmental delays, but that category, social emotional, is his strongest one. It’s also the one we can be experts in. We need PT to come in and teach us how to do PT, but to love him and show him how to express love is something that we can do. I feel like that’s probably been our most successful element”. “I think keeping your appointments, asking a lot of questions, and to just keep trying. Now he can eat, and he still doesn’t eat a lot. But the fact that he can eat and not choke is amazing. Just keep loving on that baby, he’ll surprise you”. “he’s definitely growing, and proportional. He has meat on his legs, which you know, he was only two and a half pounds when he was born. So literally he was born with rolls of skin on his inner thighs. Now he’s managed to fill those out, and he has little thighs I can actually clean. So those are the successes I see for my baby”. “I think my comfort level with caring for him, and seeing his growth, and just his personality come through, like seeing him grow. Besides the oxygen and the G-tube that he’s connected to, and having to go to so many doctor’s appointments, he is a regular baby. Now that he’s able to eat and drink by mouth, that’s huge because you want this G-tube to come out as soon as possible…The great success for me is just to see him growing to a normal baby in so many other ways, and for him to actually have started drinking from the bottle and eating food…” “He’s sitting up by his self, which, being in the hospital for so long, he had no core muscles at all. He was very loose. And now he can actually sit up on his own. So that, that to me is probably the huge success He’s full of energy and he loves to jump. Like he will jump all day long if you let him. He wants to jump every time you hold him, in your lap, almost anywhere. We have this jumper for him that he loves. He sees it, he’ll just stare at it until you put him in it. He’ll jump for so long. Like his head will get heavy and will be tilted to the side, and his body will be tilted, his legs will still be jumping on the bottom. It’s the cutest thing ever”. I think it was just us being worried because [of] what he went through. But they told me to relax, that he was okay. About when he was like two, three weeks here at home, I saw he was doing better, that’s when I started relaxing and sleeping a little bit more. And then once I brought him home and took him to his appointment that he was doing good, I’m just like okay probably I just need to relax a little bit now”.	“I think sometimes we focus a lot on all the medical problems that the baby has and kind of lose sight, and I think the providers are guilty of this too, lose sight of the developmental progress that the baby should be making at any given age, and how to support the baby as best we can”. “I’m trying to envision [what resources to offer]. I wonder if there should be two parts to this. One being the progress my child is making while hospitalized. And secondly, at discharge, what are the things I need to have? So those might be two separate things. It helps parents be really engaged and involved”.

## Data Availability

The data presented in this study are available on request from the corresponding author.
